# Supplementary material for: Improved diagnostic prediction of the pathogenicity of bloodstream isolates of Staphylococcus epidermidis
Source: PLoS One. 2021 Mar 26;16(3):e0241457. doi: 10.1371/journal.pone.0241457 (PMC7997010; doi:10.1371/journal.pone.0241457)
Supplement: S1 File — Example growth curve analysis; S1 Table. Gompertz parameters; S2 Fig OD600 vs CFUs; S3 Fig ANOSIM; S2 Table. PCR primers; S4 Fig Comparison of genotypic vs phenotypic measures methicillin resistance. (PDF) [file pone.0241457.s001.pdf]

Improved diagnostic prediction of the pathogenicity of bloodstream isolates of *Staphylococcus epidermidis*

VanAken, Shannon M, Newton, Duane, and VanEpps, J. Scott

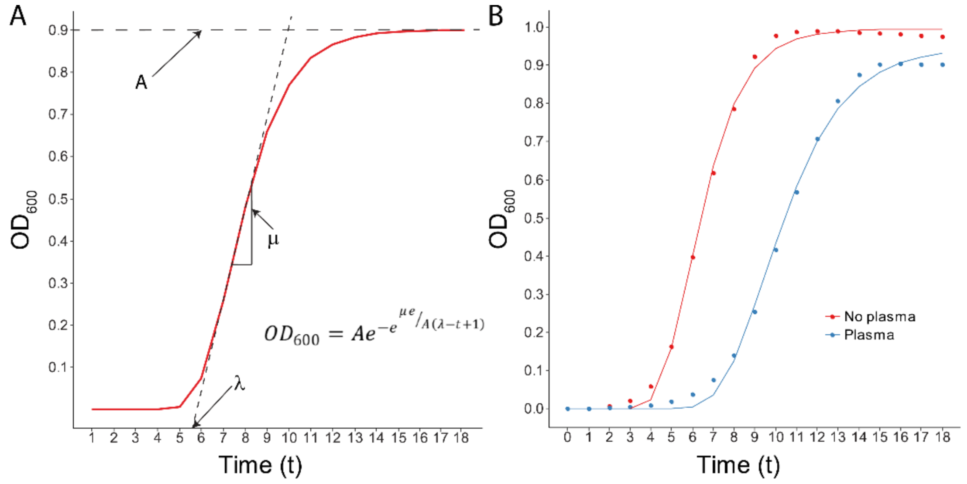

S1 Fig. Example growth curve analysis. **(A)** Theoretical Gompertz function with formula. A represents the maximum OD_{600}, μ represents the maximum growth rate, and λ represents the lag time. **(B)** Example isolate growth curve data with and without plasma supplementation and representative Gompertz fits.

S1 Table. Gompertz parameters. Determined from the Gompertz fits in **S1B Fig** as well as the ratios (Plasma:No plasma).

	A	μ (hrs^{-1})	λ (hrs)
Plasma	0.948	0.166	7.37
No plasma	0.995	0.259	4.45
Ratio (Plasma : No plasma)	0.952	0.641	1.66

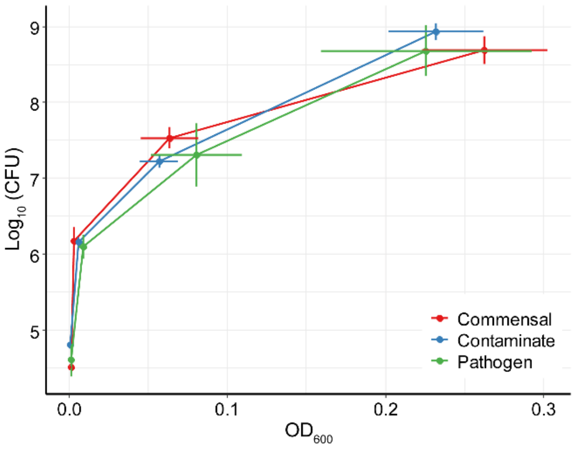

S2 Fig. OD600 vs CFUs. Comparison of the relationship between OD600 and CFUs for the exponential portion of the growth curve for a sampling of isolates from each group. Error bars represent mean ± standard deviation.

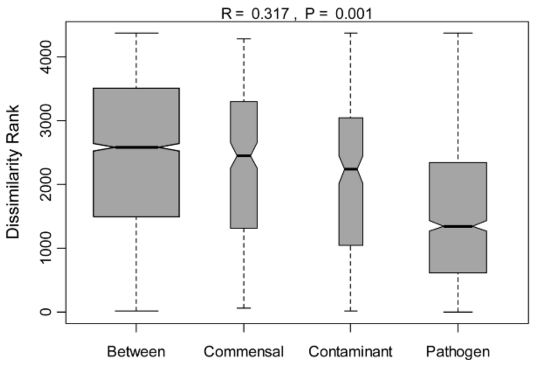

S3 Fig. **ANOSIM** comparing isolate groups. Note that the dissimilarity between isolates types was higher than that within each type but quite similar to that of the commensal and contaminates types. The pathogen type had significantly less dissimilarity indicating the possibility that this isolate type is a more homogenous subgroup.

S2 Table. PCR primers

	Forward (5'→3')	Reverse (5'→3)	Product Length	Source
mecA	AAGCGACTTCACATCTATTAGGTTAT	TATATTCTTCGTTACTCATGCCATAC	402 bp	This study
sdrF	TTCTATCCAACAAGGTAAAC	GCTGATTCTAATTGATTGTC	139 bp	This study
sesI	GCTGATTATGTAAATGACTCAAAT	AGCTT TGTTGTTTGAGCTTC	389 bp	[1]

All

		Oxacillin	
		R	S
mecA	R	53	18
	S	1	22

Commensal

		Oxacillin	
		R	S
mecA	R	1	7
	S	1	14

Contaminant

		Oxacillin	
		R	S
mecA	R	11	5
	S	0	5

Pathogen

		Oxacillin	
		R	S
mecA	R	41	6
	S	0	3

S4 Fig. Comparison of genotypic vs phenotypic measures methicillin resistance. 2x2 confusion matrices for mecA gene presence (R) vs phenotypic oxacillin resistance (R) for all isolates and separated by type.

References:

[1] B. Söderquist, M. Andersson, M. Nilsson, Å. Nilsdotter-Augustinsson, L. Persson, Ö. Friberg, S. Jacobsson, Staphylococcus epidermidis surface protein I (SesI): a marker of the invasive capacity of S. epidermidis?, J Med Microbiol 58(Pt 10) (2009) 1395-1397.
