# Supplementary material for: Improved diagnostic prediction of the pathogenicity of bloodstream isolates of Staphylococcus epidermidis
Source: PLoS One. 2021 Mar 26;16(3):e0241457. doi: 10.1371/journal.pone.0241457 (PMC7997010; doi:10.1371/journal.pone.0241457)
Supplement: S1 Table — (PDF) [file pone.0241457.s002.pdf]

S1 Table. Dataset for predictive modeling

		Antibiotic susceptibility										Growth curve parameters			Virulence genes			
Isolate ID	Type	Erythromycin	Penicillin G	Ciprofloxacin	Vancomycin	Oxacillin	Sulfanamides	Ampicillin	Clindamycin	Doxicycline	Norm CV (A_{570})	RA	Rλ	Rμ	*mecA*	*sdrF*	*ses1*	MLST
C1	Contaminant	R	R	R	S	R	R	R	R	R	0.193	0.999	1.092	0.935	+	+	-	242
C10	Contaminant	R	R	S	S	S	S	R	I	S	0.113	0.993	1.774	0.465	-	+	-	484
C14	Contaminant	S	R	R	S	S	R	R	R	S	0.125	0.990	1.071	0.783	-	+	-	57
C16	Contaminant	R	R	S	S	I	R	R	R	S	0.702	0.948	1.208	0.803	+	+	-	173
C18	Contaminant	R	R	S	S	S	S	R	R	R	0.148	0.844	1.516	0.529	+	+	-	485
C19	Contaminant	R	R	R	S	R	R	R	R	S	0.160	0.942	1.413	0.760	+	-	-	5
C20	Contaminant	R	R	S	S	I	S	R	I	R	1.095	0.990	1.191	0.924	+	+	-	NA
C22	Contaminant	R	R	R	S	R	R	R	R	I	0.244	1.021	1.147	0.808	+	-	-	242
C24	Contaminant	R	R	R	S	R	I	R	R	I	0.196	1.028	1.080	0.728	+	+	-	16
C26	Contaminant	I	R	R	S	S	R	R	S	S	0.156	0.904	1.555	0.572	-	+	-	19
C29	Contaminant	R	R	R	S	R	R	R	R	S	0.189	1.085	1.323	0.799	+	+	-	5
C36	Contaminant	R	R	R	S	R	R	R	R	S	0.158	0.950	0.992	0.619	+	+	+	2
C38	Contaminant	S	R	S	S	S	S	R	S	S	0.158	0.918	1.301	0.757	+	+	-	2
C4	Contaminant	I	R	I	I	R	I	R	R	R	0.182	0.960	1.151	0.673	+	+	-	130
C41	Contaminant	R	R	S	S	R	S	R	I	S	1.157	1.031	1.175	1.003	+	+	-	2
C43	Contaminant	S	R	S	S	S	S	R	I	S	0.117	0.962	1.345	0.875	+	+	-	59
C44	Contaminant	S	R	S	I	S	S	R	R	R	0.152	0.915	1.246	0.748	+	+	-	NA
C45	Contaminant	S	R	S	I	S	S	R	R	R	0.171	0.957	1.390	0.756	-	+	-	2
C5	Contaminant	R	R	S	S	S	I	R	R	I	0.198	0.978	1.918	0.503	-	+	-	190
C6	Contaminant	R	R	R	S	R	R	R	R	S	0.272	0.951	1.128	0.499	+	+	+	2
C7	Contaminant	S	R	S	S	S	S	R	R	S	0.515	1.159	1.237	0.876	+	-	+	5
P1	Pathogen	I	R	R	S	R	R	I	R	S	0.061	1.024	1.235	0.823	+	+	-	83
P10	Pathogen	R	R	R	S	R	R	R	R	S	0.187	0.873	1.176	0.739	+	+	+	2
P11	Pathogen	R	R	R	I	R	R	R	R	S	0.299	0.998	1.183	0.977	+	+	-	2
P12	Pathogen	R	R	R	I	R	R	R	R	S	0.194	1.050	1.083	0.788	+	+	-	210
P13	Pathogen	R	R	R	S	R	R	R	R	S	0.435	1.020	0.971	0.968	+	+	-	2
P14	Pathogen	R	R	R	S	R	R	R	R	S	0.798	1.062	0.893	0.965	+	+	-	2
P15	Pathogen	R	R	S	S	R	R	R	S	S	0.098	1.040	1.305	1.112	+	+	-	NA
P16	Pathogen	R	R	R	S	R	S	R	R	S	0.135	0.996	0.832	0.961	+	+	-	20
P17	Pathogen	R	R	S	S	R	R	R	R	S	0.060	0.998	2.312	0.592	+	+	-	NA
P18	Pathogen	R	R	R	S	R	R	R	R	S	0.089	0.889	0.916	0.590	+	+	-	2
P19	Pathogen	R	R	R	S	R	R	R	R	S	0.143	1.010	1.070	0.654	+	+	-	NA
P2	Pathogen	S	R	R	S	R	R	R	R	S	0.062	1.071	1.219	0.796	+	+	-	83
P20	Pathogen	R	R	S	S	S	R	R	R	S	0.135	1.001	0.899	0.806	+	+	-	476
P21	Pathogen	R	R	S	I	S	S	R	R	S	0.106	0.980	0.882	1.159	+	+	-	478
P22	Pathogen	R	R	S	S	R	S	R	R	S	0.300	0.993	1.056	0.919	+	+	-	59
P23	Pathogen	S	S	S	I	S	S	S	R	S	0.140	1.014	0.841	1.079	+	+	-	477
P24	Pathogen	R	R	R	S	R	S	R	R	S	0.778	0.965	1.140	0.690	+	+	-	2
P25	Pathogen	R	R	R	I	R	S	R	R	S	0.670	1.033	0.961	0.960	+	+	-	2
P26	Pathogen	I	R	S	I	I	S	R	R	S	0.267	1.084	1.178	0.696	+	+	-	130
P27	Pathogen	I	R	S	I	S	S	R	R	I	0.204	0.841	1.111	0.736	-	+	-	218
P28	Pathogen	I	R	I	S	R	S	R	I	S	0.335	1.143	0.958	1.021	+	+	-	130
P29	Pathogen	I	R	R	S	R	R	R	R	R	0.195	0.942	1.083	0.928	+	+	-	5
P3	Pathogen	R	S	R	S	S	I	R	R	S	0.057	1.027	1.134	0.895	+	+	-	425
P30	Pathogen	R	R	R	S	I	R	R	S	S	0.151	0.991	1.107	0.917	+	+	-	59
P31	Pathogen	R	R	R	S	S	R	R	R	R	0.110	0.892	1.287	0.595	+	+	-	NA
P32	Pathogen	R	R	S	I	S	R	R	R	R	0.571	1.049	1.098	0.935	+	+	-	16
P33	Pathogen	R	R	R	S	R	R	R	R	R	0.109	0.938	1.521	0.751	+	-	-	5
P34	Pathogen	R	R	R	S	R	R	R	R	S	0.122	0.918	1.565	0.738	+	+	-	5
P35	Pathogen	R	R	R	S	R	R	R	R	S	0.501	1.023	1.130	0.916	+	+	-	2
P36	Pathogen	R	R	R	S	R	R	R	R	R	0.103	0.957	1.644	0.752	+	+	-	5
P37	Pathogen	R	R	S	S	R	S	R	R	S	1.096	1.194	1.174	1.314	+	+	-	35
P38	Pathogen	R	R	S	R	S	S	R	R	R	0.905	1.000	2.134	0.613	-	+	-	167
P39	Pathogen	R	R	R	I	R	R	R	R	I	0.134	0.929	1.581	0.730	+	+	-	5
P4	Pathogen	R	R	R	S	R	R	R	R	S	0.473	1.080	1.695	0.853	+	+	-	172
P40	Pathogen	R	R	S	I	R	S	R	R	R	0.211	0.970	1.144	0.880	+	+	-	59
P41	Pathogen	R	R	R	I	R	R	R	R	S	0.912	0.949	1.076	0.895	+	+	-	2
P42	Pathogen	R	R	R	S	R	R	R	R	S	0.310	1.022	1.120	0.944	+	+	-	2
P43	Pathogen	R	R	R	S	R	R	R	R	S	0.965	0.829	1.093	0.519	+	+	+	2
P44	Pathogen	R	R	R	S	R	R	R	R	S	0.718	0.905	1.109	0.928	+	+	-	5
P45	Pathogen	R	R	R	S	R	R	R	R	S	0.108	0.777	1.792	0.471	+	+	-	5
P46	Pathogen	S	R	R	I	R	R	R	I	S	0.890	0.977	1.144	0.835	+	+	-	83
P47	Pathogen	R	R	R	S	R	S	R	S	S	1.019	1.057	0.967	1.022	+	+	-	69
P48	Pathogen	R	R	R	I	R	R	R	R	S	0.972	0.976	1.181	0.991	+	+	-	2
P49	Pathogen	R	R	S	S	S	S	R	S	S	0.144	1.005	1.173	0.841	-	+	-	17
P5	Pathogen	S	S	S	S	I	S	S	R	S	0.163	1.088	1.899	0.969	+	+	-	83

S1 Table. Dataset for predictive modeling

P50	Pathogen	R	R	S	S	S	S	R	S	S	0.042	1.004	1.174	0.857	+	+	-	17
P51	Pathogen	R	R	R	I	R	R	R	R	S	0.918	0.951	1.245	0.923	+	+	-	2
P52	Pathogen	R	R	R	S	R	R	R	R	S	0.100	1.046	1.675	0.711	+	+	-	110
P53	Pathogen	R	R	S	S	R	S	R	R	S	0.127	0.919	1.825	0.638	+	+	-	5
P54	Pathogen	I	R	S	S	R	S	R	R	S	0.527	0.956	1.250	0.838	+	+	-	297
P6	Pathogen	S	R	R	S	I	R	R	S	S	1.014	1.125	1.503	0.929	+	+	-	83
P7	Pathogen	S	R	R	S	R	S	R	S	S	0.124	1.035	1.862	0.721	+	+	-	5
P8	Pathogen	I	R	R	S	R	R	R	S	S	0.442	0.999	1.261	0.838	+	+	-	2
P9	Pathogen	R	R	R	S	R	R	R	R	S	0.097	0.870	1.986	0.507	+	+	-	5
S1	Commensal	I	R	S	S	S	S	R	R	S	0.116	1.025	1.425	0.642	-	+	-	190
S12	Commensal	S	R	S	S	S	S	R	S	S	0.151	1.084	1.502	1.008	+	+	-	136
S13	Commensal	R	R	S	S	S	S	R	I	S	0.064	1.089	1.800	0.670	+	+	-	482
S14	Commensal	R	R	R	S	S	R	R	R	S	0.426	0.992	1.231	0.876	+	+	-	2
S15	Commensal	S	S	S	S	S	S	R	I	S	0.132	0.944	1.480	0.558	-	+	-	6
S16	Commensal	S	R	S	S	S	R	R	I	S	0.096	1.043	1.219	0.926	-	+	-	89
S18	Commensal	R	R	S	S	S	R	R	R	S	0.061	0.882	2.155	0.442	+	-	-	481
S19	Commensal	S	R	S	S	S	S	R	S	S	0.618	1.020	0.965	1.123	-	+	-	35
S2	Commensal	R	R	S	S	R	R	R	R	S	0.052	1.017	1.641	0.603	-	-	-	422
S20	Commensal	R	R	S	S	S	S	R	I	S	0.188	0.934	2.771	0.444	-	+	-	72
S21	Commensal	S	R	S	S	S	S	R	I	S	0.368	1.167	1.030	1.119	+	+	-	475
S22	Commensal	S	R	S	I	S	S	R	I	S	0.080	0.980	1.273	0.846	-	-	-	5
S23	Commensal	R	R	S	S	S	S	R	S	S	0.296	1.021	1.043	0.905	-	+	-	35
S24	Commensal	S	R	S	S	S	S	R	R	S	1.057	1.040	1.334	0.640	-	+	-	479
S25	Commensal	S	R	S	S	S	S	R	I	S	0.050	1.128	1.192	0.850	-	+	-	480
S3B	Commensal	R	R	R	S	R	S	R	S	S	0.081	0.949	1.749	0.657	+	+	-	73
S3C	Commensal	S	S	S	S	S	S	S	R	S	0.053	1.074	1.140	0.993	-	+	-	110
S4	Commensal	R	R	S	S	S	S	R	I	S	0.223	0.981	1.918	0.707	-	-	-	73
S5	Commensal	R	R	S	S	S	S	R	I	S	0.145	1.061	1.906	0.767	-	-	-	256
S6	Commensal	I	R	S	S	S	S	R	I	S	0.054	0.952	2.427	0.614	-	+	-	423
S7	Commensal	R	S	S	S	S	S	S	I	S	0.189	0.901	1.807	0.654	+	-	-	73
S8	Commensal	I	R	S	S	S	S	R	R	S	0.123	0.932	1.603	0.769	-	+	-	73
S9	Commensal	I	R	S	S	S	S	S	I	S	1.021	1.087	1.926	0.685	+	+	+	424
